# Investigating the effectiveness of monitoring relevant variations during IMRT and VMAT treatments by EPID-based 3D *in vivo* verification performed using planning CTs

**DOI:** 10.1371/journal.pone.0218803

**Published:** 2019-06-28

**Authors:** Yinghui Li, Jinhan Zhu, Jinping Shi, Lixin Chen, Xiaowei Liu

**Affiliations:** 1 The First People’s Hospital of FoShan (Affiliated FoShan Hospital of Sun Yat-sen University), Foshan, Guangdong, China; 2 State Key Laboratory of Oncology in South China, Sun Yat-sen University Cancer Center, Sun Yat-Sen University of Medical Sciences, Guangzhou, Guangdong, China; 3 School of Physics, Sun Yat-sen University, Guangzhou, Guangdong, China; University of Seville, SPAIN

## Abstract

**Purpose:**

The goal of this study was to investigate the effectiveness of monitoring relevant variations during treatments for electronic portal imaging device (EPID)-based 3D in vivo verification performed using planning CTs.

**Methods:**

Experiments on two simple phantoms (uniform and nonuniform phantoms) and a thoracic phantom were analyzed in this study, and six relevant variations including the machine output, planning target volume (PTV) deformation, multileaf collimator (MLC) and Phantom shift (set-up errors), and gantry and couch angle shifts were evaluated. 3D gamma and dose-volume histogram (DVH) methods were used to evaluate the detection sensitivity of the EPID-based 3D in vivo dosimetry and the dose accuracy of the EPID reconstruction, respectively, as affected by the variations, and the results were validated by determining the consistency with TPS simulated results.

**Results:**

The results of the simple phantoms showed that the gamma failure rates and DVH trend of EPID reconstructions were consistent with the results of TPS simulations for machine output and MLC shifts and inconsistent for phantom shift, gantry/couch angle shift and PTV deformation variations. The results of the thoracic phantom showed that CBCT-guided EPID reconstruction sensitively detected 3-mm Phantom shift in thoracic phantom and its gamma failure rates and DVH trend were consistent with the results of TPS simulations.

**Conclusion:**

The variations, such as machine output and MLC shift, that are phantom unrelated and cause changes in the beam of the linear accelerator can be sensitively detected by EPID-based 3D in vivo dosimetry and do not affect the accuracy of the EPID reconstruction dose. Planning CT will limit the detection sensitivity and the accuracy of the reconstruction dose of the EPID-based 3D in vivo dosimetry for phantom-related variations (such as Phantom shift and gantry/couch angle shift). EPID reconstruction combined with IGRT technology is a more effective method to monitor phantom shift variations.

## Introduction

Advanced radiation therapy technologies such as intensity-modulated radiation therapy (IMRT) and volumetric-modulated arc therapy (VMAT) have been widely used in clinical practice because of their superior tumor dose conformity and reduced radiation to at-risk organs. Because of the complexity of these treatment techniques, patient-specific dose verification has been widely recommended to avoid major treatment errors [1]. This verification is primarily conducted prior to treatment using 2D/3D dosimetry [2]. This pretreatment verification can detect errors such as the wrong plan file and poor machine performance (MLC and output errors, among others) before the initiation of the treatment [3]. However, variations during the clinical treatment cannot be detected using pretreatment verification. Therefore, in vivo dose verification as a measurement method during treatment has attracted increasing attention worldwide.

Electronic portal imaging devices (EPIDs) are often utilized for pretreatment and in vivo dose verification because of their favorable characteristics, such as a high resolution, real-time response, digital format and low workload [3–7]. EPIDs are becoming increasingly popular for in vivo dose verification because the EPID images can be used to reconstruct a 2D plane or 3D dose distribution within the patient CTs by means of some type of reconstruction algorithm [8–10]. Cone-beam computed tomography (CBCT), as online images acquired in the course of treatment, can effectively reflect the position errors and anatomical changes of patients. Thus, CBCT images seems to be the best choice for EPID-based in vivo verification, and a few studies have also proven the feasibility of CBCT selection [11, 12]. However, the image quality of CBCT is poor, and it needs to be calibrated before being used for dose calculation, which limits the application of CBCT to EPID-based in vivo dose verification.

There have also been some studies directly using planning CTs for EPID-based in vivo dose verification in clinical treatment [13–17]. Mans et al. [16] reported that 17 serious errors were detected among the treatment plans of 4337 patients, and Bojechko et al. [17] reported that by combining all of the verification steps, 91% of all external beam therapy incidents could be detected. Although much clinical experience indicates that EPID-based in vivo verification performed using planning CTs is an efficient method of patient-specific dose verification, it is still controversial because variations in the patient positioning and/or patient anatomy during treatment (due to deformation, changing air gaps or weight loss) may result in an unreliable estimate of the in vivo verification results. It is thus necessary to verify the rationality of choosing planning CT for EPID-based in vivo dose reconstruction including the sensitivity of the variation detection and the effect of the variation on the dose reconstruction.

Bojechko et al. [8] investigated the sensitivity of EPID-based 2D in vivo verification for relevant errors in treatment delivery by evaluating gamma pass rates. The results indicate that EPID-based 2D in vivo verification can detect relatively small variations in the overall dose and systematic shifts of the MLCs, but changes in the patient’s habitus and shifts in the patient’s position were not readily detected. Compared with 2D in vivo verification, EPID-based 3D in vivo verification can reflect the dose difference of each organ structure. Therefore, it is necessary to investigate the sensitivity of EPID-based 3D in vivo verification.

Several studies have reported the feasibility of using EPID-based 3D in vivo verification as a dose-guided radiotherapy (DGRT) method [18, 19]. Therefore, the accuracy of the reconstruction dose is very important. Numerous reports of EPID-based dose reconstruction algorithms have appeared [20–22]. However, to our knowledge, no study has appeared to date that discusses the effect of variations on the accuracy of the reconstruction dose.

The purpose of this work was to evaluate the detection sensitivity of EPID-based 3D in vivo dosimetry performed using planning CTs and the effect of variations on the accuracy of the EPID reconstruction dose. Two simple phantoms and a thoracic phantom were used, and six relevant variations including the machine output, PTV deformation, MLC and Phantom shifts, and gantry and couch angle shifts were evaluated in this work. 3D Gamma and DVH methods were used to evaluate the detection sensitivity of EPID-based 3D in vivo dosimetry and the dose accuracy of the EPID reconstruction, respectively, as affected by these variations, and the results were validated by determining the consistency with TPS simulated results.

## Materials and methods

### Phantom and treatment planning

Two 30 × 30 × 16 cm^3^ simple phantoms (one uniform and one nonuniform phantom) were used to verify the effect of the uniformity of the tissue density at the exposure site on EPID-based 3D in vivo dose verification. The uniform simple phantom consists of solid water with a thickness of 16 cm, and the nonuniform simple phantom consists of 10 cm thick solid water and 6 cm thick wood. A 6 cm diameter plexiglass cylinder (1.2 g/cm3) is embedded in the wood as a planning target volume (PTV), and the lung tissue equivalent material wood (30 × 30 × 6 cm3, 0.26 g/cm3) was used as the organ at risk (OAR).

**Fig 1:**
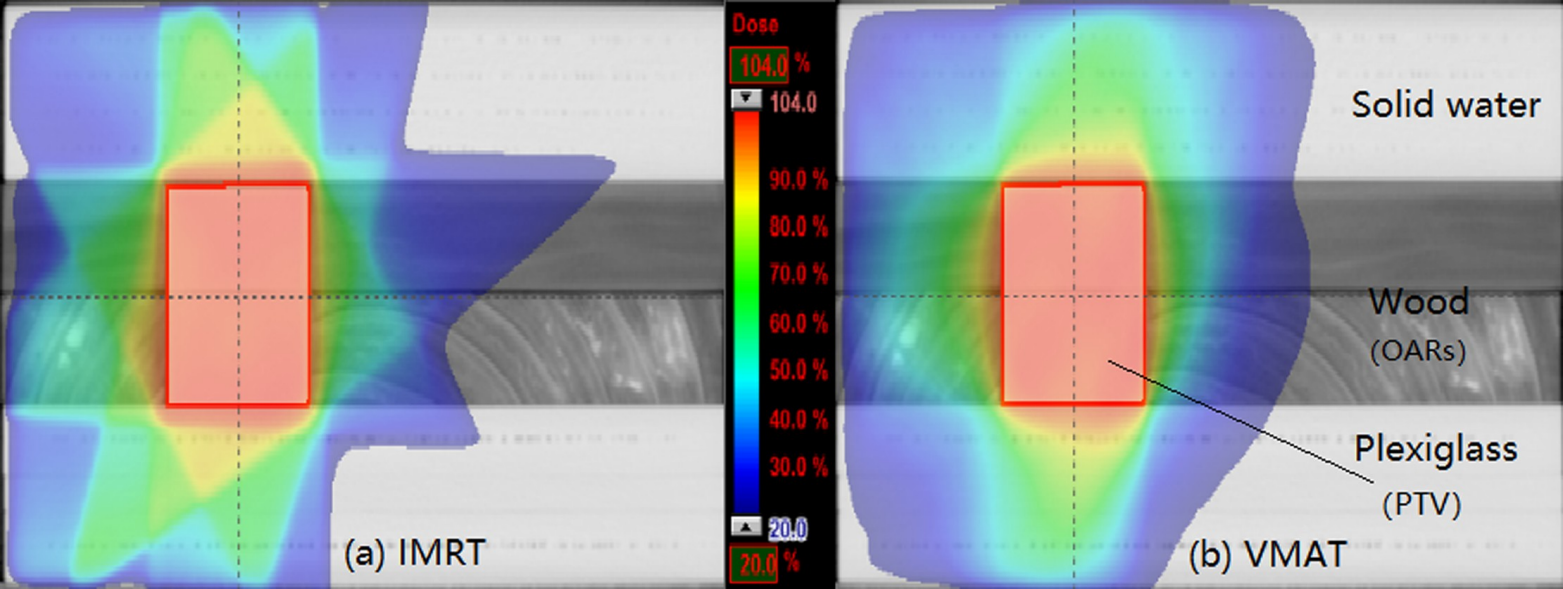
The dose distributions for the (a) IMRT and (b) VMAT plans.

One IMRT and one VMAT treatment plan were created in the nonuniform simple phantom and the prescribed dose was 50 Gy to the PTV and V20 <30% on the volume of the lung tissue equivalent material wood (OAR). The relative dose distribution is presented in Fig 1. To ensure the consistency of the treatment plan and structure in the two simple phantoms, the structures (PTV and OAR) and treatment plan in the nonuniform phantom were copied to the uniform simple phantom CT via rigid registration and a verification plan, respectively. All treatment plans were generated in the Eclipse treatment planning system v10.0.28 (Varian Medical Systems, Palo Alto, CA, USA) using the anisotropic analytical algorithm (AAA) and a 3-mm grid size for dose calculation.

To ensure that the experimental data in the simple phantoms have clinical reference value, we also validated the thoracic phantom and an actual clinical IMRT treatment plan in this study, as shown in S1 Fig. The prescribed dose was 50 Gy to the PTV with restrictions of a maximum dose of 40 Gy to a mock spinal cord and V20 <30% on the combined volume of both lungs.

### Simulation of treatment variations

For the simple phantoms, six relevant variations including the machine output, PTV deformation, MLC position and Phantom shifts (set-up errors), and gantry and couch angle shifts were introduced in the treatment plan. The parameter of each variation was adjusted three times and performed in four conditions (IMRT and VMAT in the nonuniform and uniform phantoms). A total of 4 original plans and 66 modified plans were measured in this study (the PTV deformation was measured only in the nonuniform phantom), and a detailed description of the modifications is provided in Table 1.

**Table 1:** Description of modifications for treatment plan.

Variation	Description of modifications	Plan execution condition
Machine output	Total monitor units (MUs) increased by 3%, 5% and 10%	IMRT and VMAT in the nonuniform and uniform phantoms
MLC shift	Systematic shifting of all MLC positions by 0.5, 1 and 2 mm (decreasing the gap between the MLC leaves)	
Phantom shift	Iso-center shifts of 3, 5 and 10 mm in the horizontal direction	
Gantry angle shift	Gantry angle shifts of 1°, 2° and 5°	
Couch angle shift	Couch angle shifts of 1°, 2° and 5°	
PTV deformation	The volume of the plexiglass cylinder was gradually reduced (the diameter was decreased from 6 cm to 4 cm, 2 cm and 0 cm) and replaced with wood	IMRT and VMAT in the nonuniform phantoms

Considering that the positioning shifts are patient-related, we added the thoracic phantom test to the Phantom shift on six setup directions including the left-right, superior-inferior and anterior-posterior directions. In addition, in clinical radiotherapy, the positional shifts can usually be guided by CBCT technology. Therefore, besides directly reconstructing the EPID in vivo dose in the planning thoracic phantom CT (EPID reconstruction), we also adjusted the planning CT position according to the CBCT position guided (IGRT, image-guided radiotherapy) and then reconstructed the EPID dose in the adjusted planning CT (defined as CBCT-guided EPID reconstruction).

### EPID image acquisition and dose reconstruction

A Trilogy 6 MV linear accelerator system (Varian Medical Systems, Palo Alto, CA, USA) with an aS1000 EPID (Varian Medical Systems) was employed in this study. The EPID had a sensitive area of 40 cm × 30 cm, and the effective pixel size was 0.04 cm × 0.04 cm. The effective source-to-detector distance was set to 140 cm. Image acquisition was performed with IAS3 software (Varian Medical Systems, Palo Alto, CA, USA). EPID images of the IMRT fields were captured using the integrated mode, and the VMAT fields were captured using the continuous mode. Each image was acquired with offset correction, gain correction and pixel correction.

**Fig 2:**
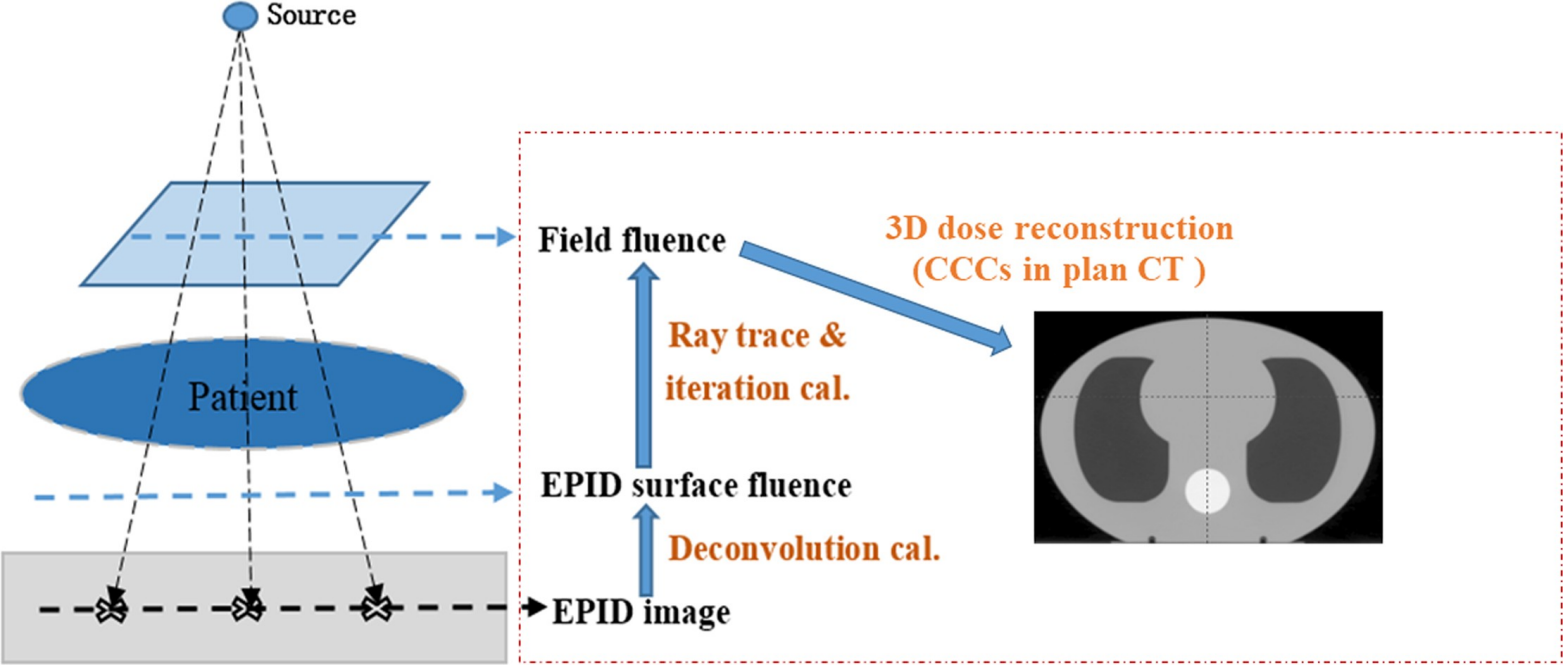
Schematic diagram of EPID-based 3D in vivo dose reconstruction.

The EPID images were imported into an in-house software for dose reconstruction, and a detailed description of the algorithm is presented in Refs. [22] and [23]. First, the EPID images were deconvolved and convoluted by the EPID panel-specific fitted kernel to obtain the EPID surface fluence; then, the field fluence was computed using the EPID surface fluence maps with the ray trace and iteration calculation. Finally, the 3D in vivo dose was computed using field fluence maps with the collapsed-cone convolution/superposition (CCCS) algorithm in planning CTs. The calculation grid size was 3 mm to match that of the Eclipse TPS. A schematic diagram of the EPID-based 3D in vivo dose reconstruction is shown in Fig 2.

### Evaluation method

To avoid the interference of the different algorithms, the EPID reconstruction doses of the modified plans were directly compared with the EPID reconstruction dose of the original plan, and all comparisons are simulated by TPS for consistency verification.

The sensitivity of the EPID-based 3D in vivo verification was evaluated by the 3D gamma method implemented with independent calculation. A global 3D gamma analysis was performed for the whole phantom (Body) and PTV structures. The parameters of the 3D gamma index were a 3% dose difference and a 3-mm distance (3%/3 mm) with a consistency of at least a 95% pass rate and criteria of 2%/2 mm with a consistency of at least a 90% pass rate [24, 25]; a cut-off dose of 10% of the maximum dose was used. For easier comparison and a better overview, the sensitivity results are presented as the percentage of the measuring points with a gamma value above 1 (γ>1), herein called the gamma failure rate [26], and the consistency with the TPS simulation results was evaluated.

Similar to the treatment plan, the EPID-based 3D in vivo verification can also provide DVH information for reconstruction quality assessment. In this study, the dose difference between the modified and original plans was evaluated using the DVH evaluation method, and then the dose accuracy of EPID reconstruction was verified by a consistency comparison with the DVH dose difference simulated by TPS.

## Results

### Sensitivity analysis for simple phantom

**Fig 3:**
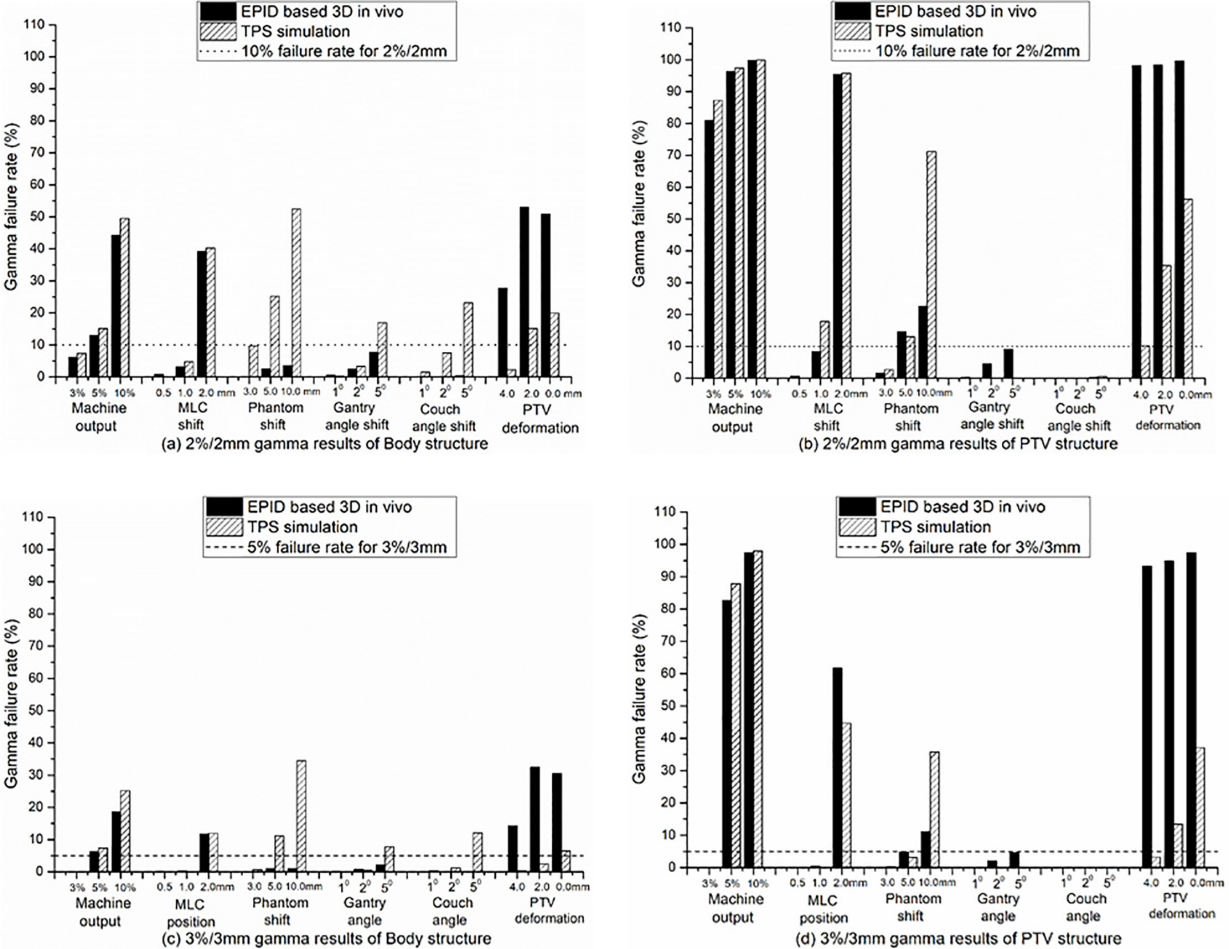
Gamma consistency comparison between EPID reconstruction and TPS simulation. (a) and (b) 2%/2 mm gamma results for Body and PTV structures, respectively. (c) and (d) 3%/3 mm gamma results for Body and PTV structures, respectively. The results were the average of the IMRT and VMAT plan results in both nonuniform and uniform simple phantoms.

A gamma consistency comparison between the EPID reconstruction and TPS simulation is shown in Fig 3. Fig 3(A) and 3(B) show the 2%/2 mm results for the Body and PTV structures, respectively, and Fig 3(C) and 3(D) show the 3%/3 mm results. The gamma results of the machine outputs and MLC shift show that the sensitivity of EPID reconstruction is consistent with the TPS simulation. However, other variations including the Phantom shift, gantry and couch angle shifts and PTV deformation show that the sensitivity of the EPID reconstruction is not consistent with the TPS simulation. The TPS simulation can easily detect a 5-mm Phantom shift and 5° gantry and couch angle shifts by the gamma failure rate, whereas the EPID reconstruction can only detect a 5-mm Phantom shift from the 2%/2 mm PTV results. For the PTV deformation, the results show that the sensitivity of the EPID reconstruction is significantly higher than that of the TPS simulation.

**Fig 4:**
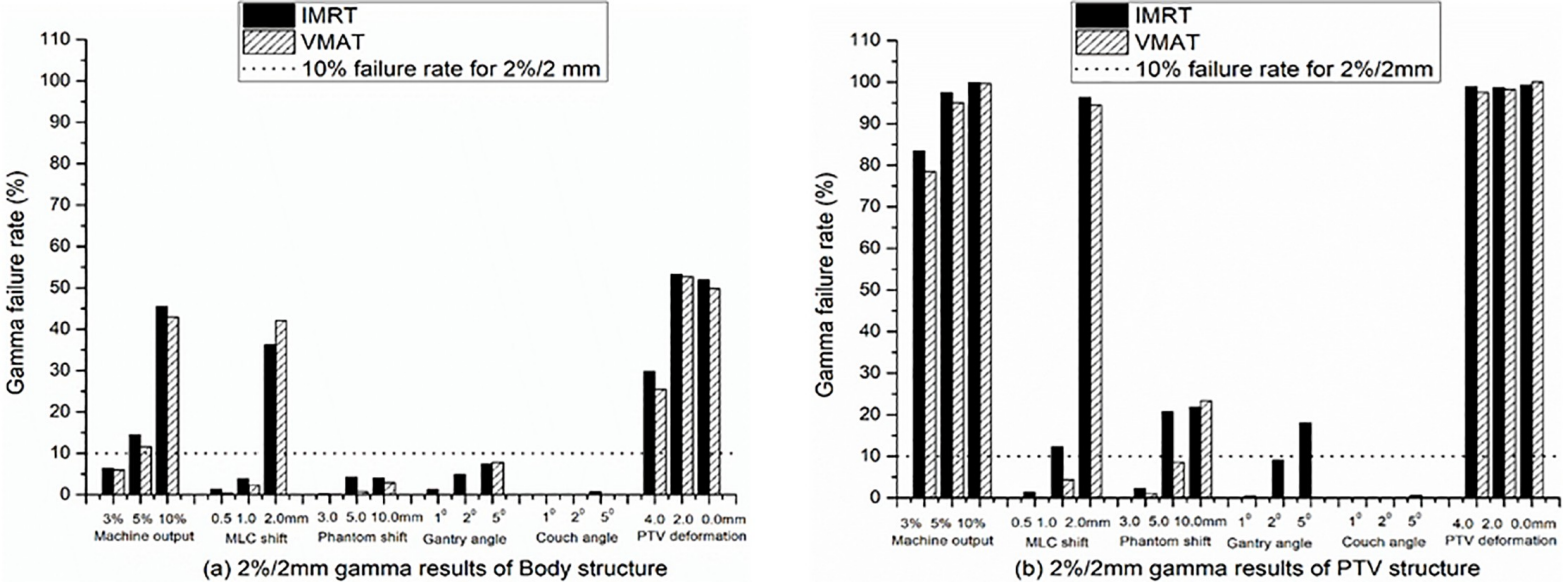
Gamma comparisons of EPID reconstruction dose between IMRT and VMAT treatment techniques. (a) and (b) 2%/2 mm gamma results for Body and PTV structures, respectively. The dotted line represents a failure rate of 10% for the 2%/2 mm criteria. The results were the average of nonuniform and uniform phantoms.

The gamma comparisons of the EPID reconstruction dose between the IMRT and VMAT treatment techniques for the Body and PTV structures are shown in Fig 4, taking the 2%/2 mm results as an example. The results show that the treatment technology had no effect on the detection sensitivity of the EPID reconstruction except for the gantry angle shift. EPID reconstruction may be more sensitive to detecting the gantry angle shift of IMRT technology than to detect the VMAT gantry angle shift (Fig 4(B)).

**Fig 5:**
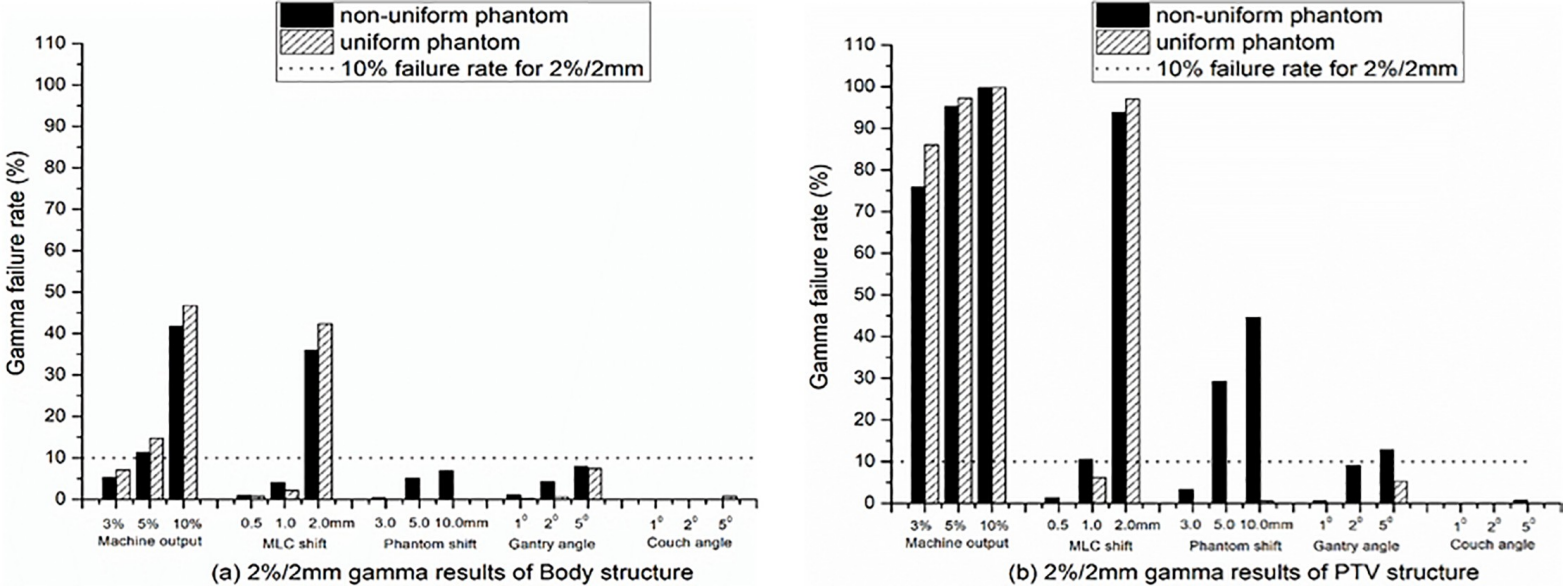
Gamma comparisons of EPID reconstruction dose between nonuniform and uniform simple phantoms. (a) and (b) for 2%/2 mm gamma results for Body and PTV structures, respectively. The gamma results are the average of the results of the IMRT and VMAT plans.

The gamma comparisons of the EPID reconstruction dose between nonuniform and uniform simple phantoms are shown in Fig 5, taking the 2%/2 mm results as an example. The results show that the detection sensitivity of the EPID reconstruction for the machine output and MLC shift was independent of the uniformity of the tissue density at the exposure site. In contrast, the sensitivity of the EPID reconstruction for the phantom and gantry angle shift displacements is strongly dependent on the uniformity of the tissue density at the exposed site, especially the gamma results of PTV (Fig 5(B)). EPID reconstruction does not show high sensitivity for the couch angle shift, whether in nonuniform or uniform simple phantoms.

### DVH analysis for simple phantom

**Fig 6:**
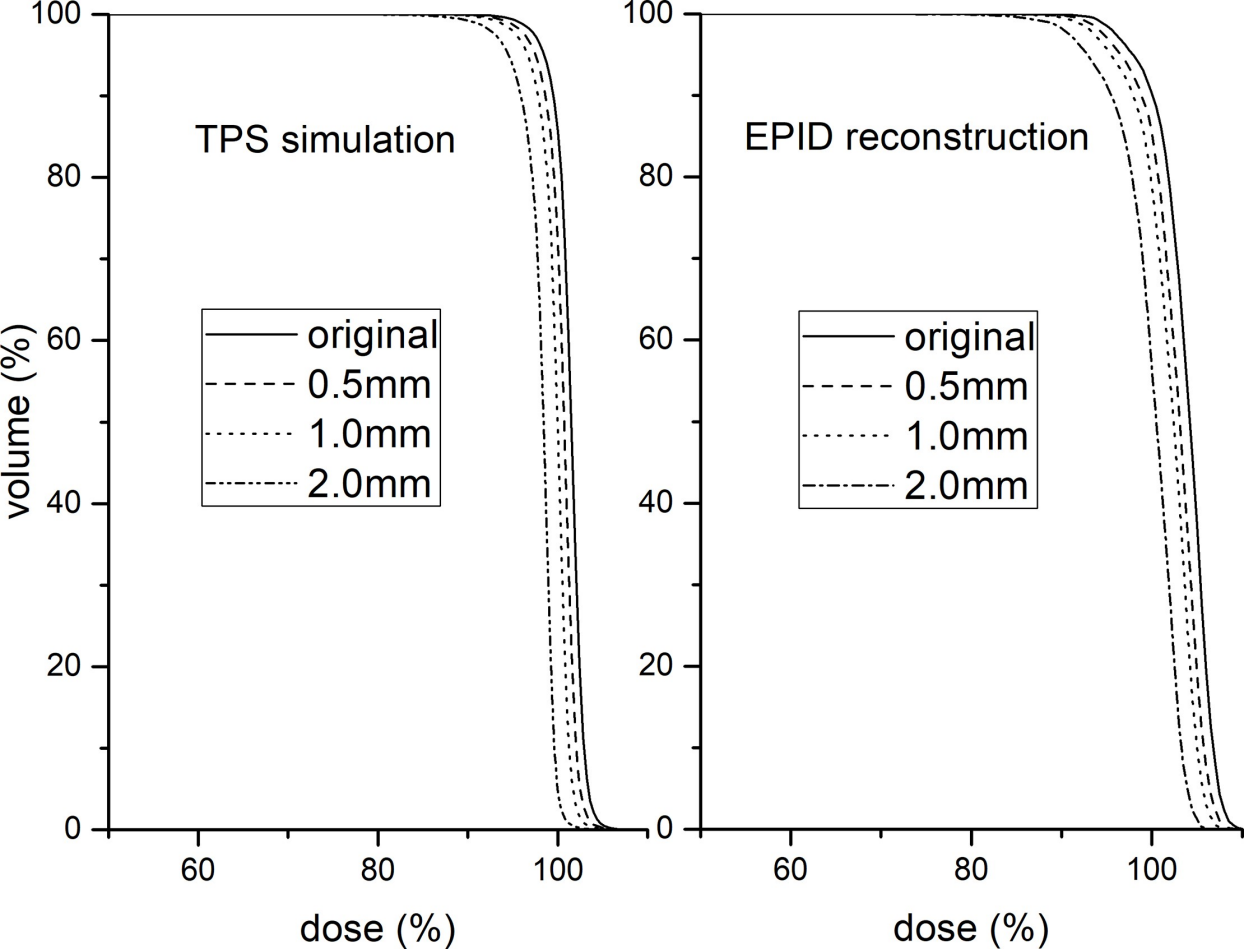
PTV DVH consistency comparison between EPID reconstruction and TPS simulation for MLC shift. TPS simulation (left). EPID reconstruction (right).

A PTV DVH consistency comparison between the EPID reconstruction and TPS simulation for the MLC shift is shown in Fig 6. It can be seen that the PTV DVH trend of the EPID reconstruction is consistent with that of the TPS simulation, which indicates that the MLC shift has no effect on the accuracy of the EPID reconstruction dose. The change ratios of the PTV DVH in the TPS simulation (Fig 6 left) and EPID reconstruction (Fig 6 right) were 2.0%/mm and 1.8%/mm, respectively. The machine output variation displayed similar results to the MLC shift, and the ratios were approximately 1:1 for both the EPID reconstruction and TPS simulation.

**Fig 7:**
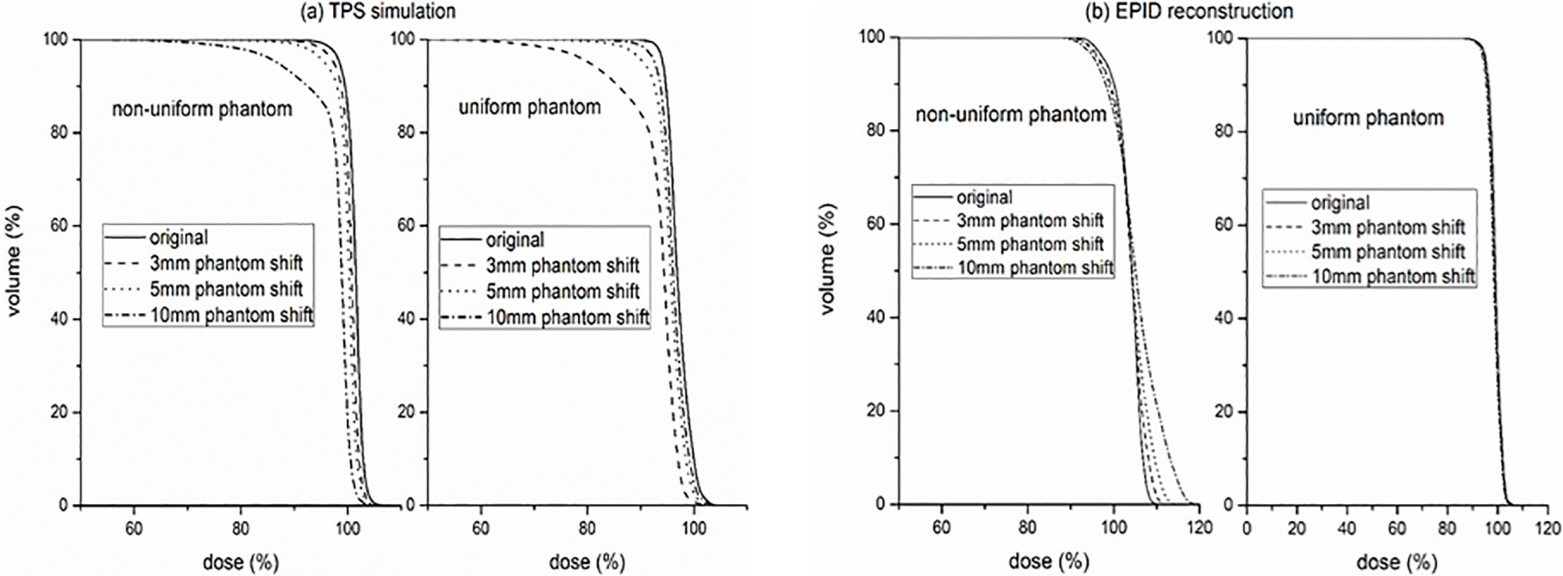
PTV DVH consistency comparison between EPID reconstruction and TPS simulation for Phantom shift, (a) TPS simulation. (b) EPID reconstruction.

The PTV DVH consistency comparison between the EPID reconstruction and TPS simulation for the Phantom shift are shown in Fig 7. The TPS simulation results (Fig 7(A)) show that the Phantom shift results in an insufficient dose of PTV DVH and is independent of the uniformity of the phantom. The EPID reconstruction results (Fig 7(B)) show that the trends of the PTV DVH change caused by the position shifts of the uniform and nonuniform phantoms are different, and both are inconsistent with that of the TPS simulation. It can be seen that the nonuniform Phantom shift causes the PTV DVH of the EPID reconstruction to have dose changes compared to the original plan (Fig 7(B) left). However, a uniform Phantom shift has little effect on the PTV DVH of the EPID reconstruction (Fig 7(B) right). Table 2 presents the relative dose differences of D95, D50 and D5 between the Phantom shift plans and the original plans.

**Table 2:** Relative dose differences of D95, D50 and D5 between the Phantom shift plans and the original plans.

		IMRT						VMAT					
		Nonuniform			Uniform			Nonuniform			Uniform		
		D95	D50	D5	D95	D50	D5	D95	D50	D5	D95	D50	D5
**TPS**	**3 mm**	-1.0%	-0.9%	-0.8%	-1.2%	-0.9%	-0.5%	-1.0%	-0.7%	-0.7%	-1.5%	-0.8%	-0.7%
	**5 mm**	-2.2%	-1.5%	-1.4%	-2.2%	-1.5%	-1.0%	-2.8%	-1.2%	-1.1%	-3.6%	-1.3%	-1.4%
	**10 mm**	-9.9%	-3.1%	-2.8%	-12.2%	-3.2%	-2.6%	-11.6%	-2.6%	-2.3%	-13.7%	-2.4%	-3.0%
**EPID**	**3 mm**	-1.3%	-0.3%	1.4%	-0.5%	-0.5%	-0.3%	-1.0%	-0.1%	1.6%	-0.2%	-0.2%	-0.2%
	**5 mm**	-3.5%	-0.2%	2.8%	-0.7%	-0.7%	-0.3%	-1.3%	0.1%	3.4%	-0.4%	-0.3%	-0.4%
	**10 mm**	-4.0%	-1.6%	4.8%	-1.2%	-1.2%	-0.3%	-2.2%	0.8%	7.5%	-1.0%	-0.8%	-0.6%

**Fig 8:**
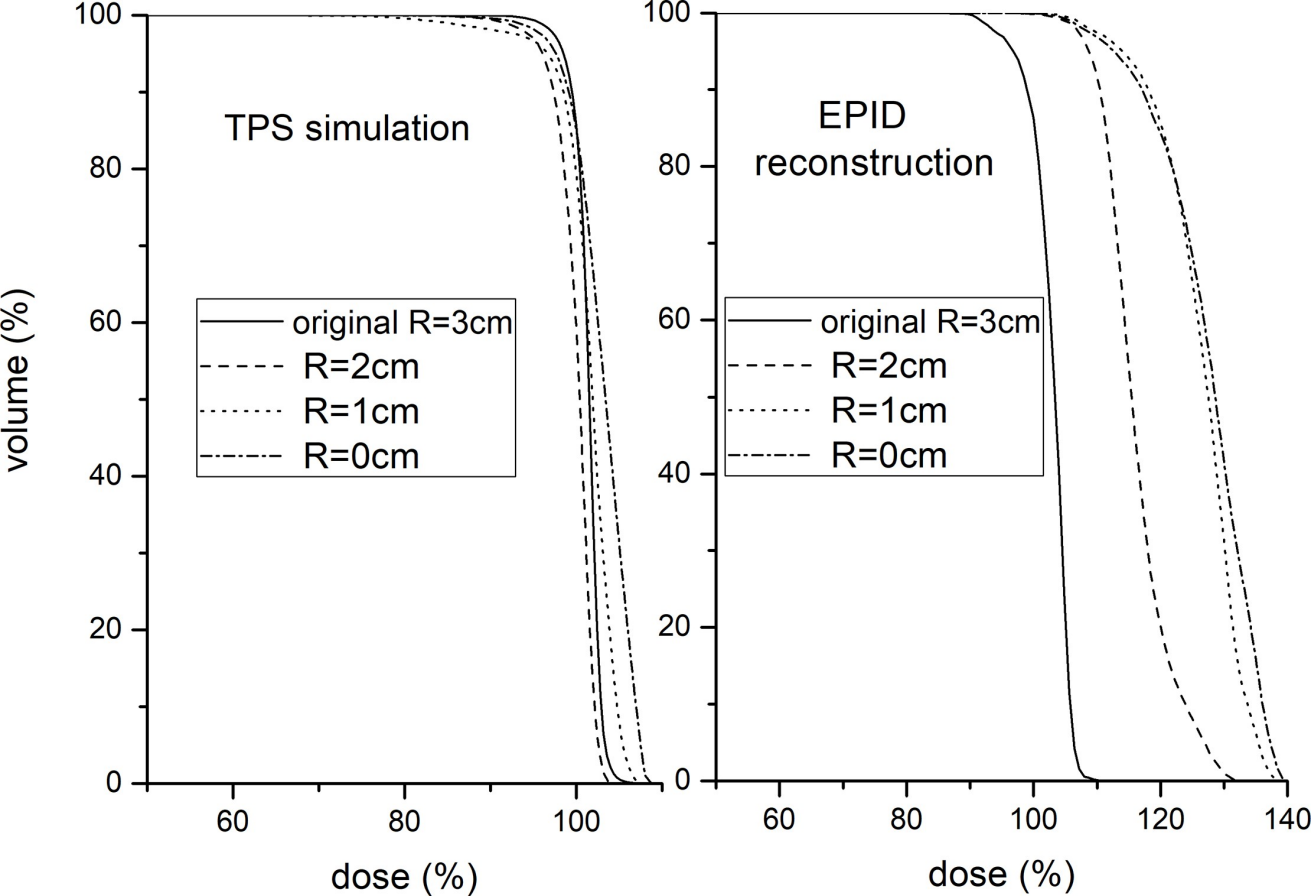
PTV DVH consistency comparison between EPID reconstruction and TPS simulation for PTV deformation. TPS simulation (left). EPID reconstruction (right).

A PTV DVH consistency comparison between the EPID reconstruction and TPS simulation for the PTV deformation is shown in Fig 8. The results show that the PTV DVH trend of the EPID reconstruction is inconsistent with that of the TPS simulation and the PTV DVH differences is more than 10%. The PTV DVH consistency comparison for the gantry and couch angle shifts was omitted because of the small dose difference from the original plan.

### Analysis for thoracic phantom

**Fig 9:**
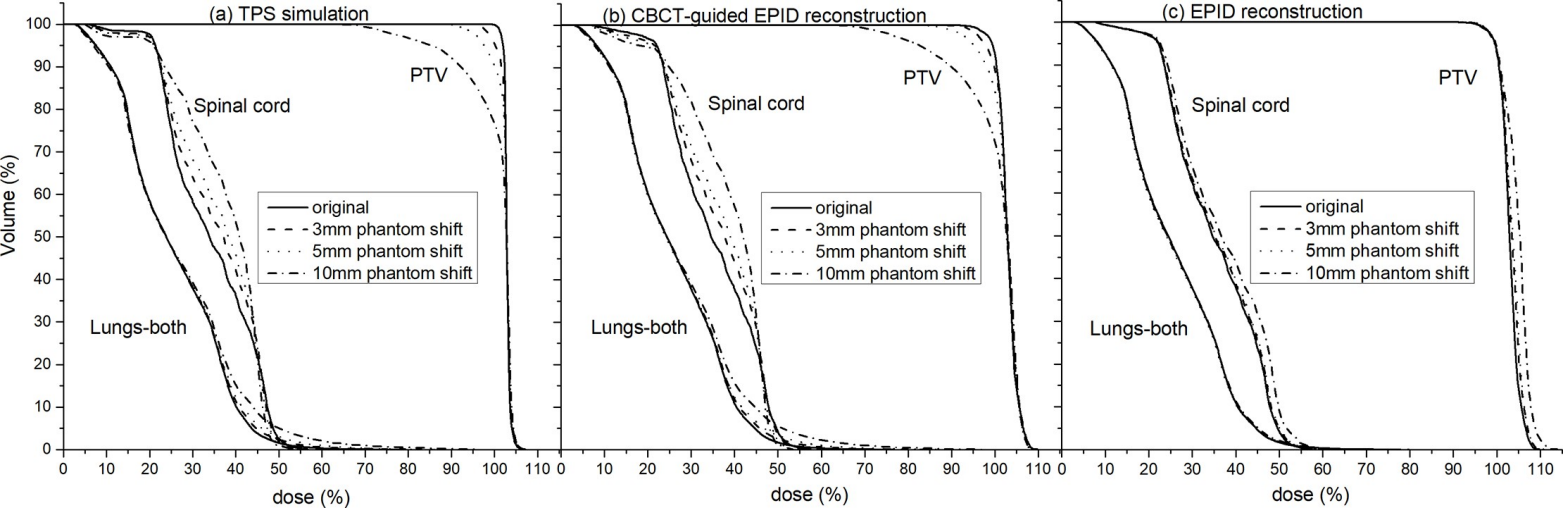
DVH consistency comparison of EPID reconstruction and CBCT-guided EPID reconstruction with TPS simulation for phantom left shift. (a) TPS simulation. (b) CBCT-guided EPID reconstruction. (c) EPID reconstruction.

For the thoracic Phantom shift, the gamma consistency comparison between the EPID reconstruction and TPS simulation is shown in Table 3. Similar to the simple Phantom shift, the gamma failure rate of the EPID reconstruction is significantly lower than that of the TPS simulation, and it is almost insensitive to 3 and 5 mm Phantom shifts. However, the CBCT-guided EPID reconstruction shows a high detection sensitivity to a 3 mm Phantom shift, and the gamma failure rate is consistent with the TPS simulation results. A DVH consistency comparison can also obtain similar results, as shown in Fig 9, with the phantom left shift as an example. It can be seen that the EPID reconstruction can only detect DVH differences (PTV and spinal cord) caused by a 10 mm thoracic Phantom shift (Fig 9C), and the trend of the DVH is not consistent with the TPS simulation results. CBCT-guided EPID reconstruction can detect DVH differences caused by a 3 mm Phantom shift, and the DVH trends of PTV and OARs (lungs and spinal cord) are consistent with the TPS simulation results (Fig 9B). The 2%/2 mm pass rate between the TPS and EPID reconstruction for the original plan was 95.4%, and the isodose and gamma distribution are shown in S2 Fig.

**Table 3:** Gamma failure rate statistics of pure EPID reconstruction, CBCT-guided EPID reconstruction and TPS simulation for thoracic Phantom shift.

		Body			PTV		
		TPS simulation	EPID reconstruction (+CBCT-guided)	EPID reconstruction	TPS simulation	EPID reconstruction (+CBCT-guided)	EPID reconstruction
Left-right direction	±3 mm	10.5%	11.6%	0.1%	5.3%	8.0%	0.3%
	±5 mm	25.2%	27.7%	0.3%	15.7%	19.0%	3.2%
	±10 mm	46.3%	51.0%	5.8%	31.5%	37.2%	43.6%
Superior-inferior direction	±3 mm	12.7%	12.5%	0.0%	7.2%	10.0%	0.0%
	±5 mm	26.4%	27.7%	0.1%	17.4%	22.3%	1.3%
	±10 mm	44.6%	47.0%	1.8%	37.6%	45.3%	18.5%
Anterior-posterior direction	±3 mm	6.4%	3.4%	0.0%	1.7%	1.4%	0.0%
	±5 mm	12.5%	7.3%	0.0%	5.3%	4.5%	0.0%
	±10 mm	27.4%	22.7%	0.0%	14.7%	13.9%	0.0%

## Discussion

In this study, the sensitivity and dose accuracy of EPID-based 3D in vivo dosimetry to variations including those in the machine output, PTV deformation, MLC position and Phantom shifts, and gantry and couch angle shifts during treatments were evaluated.

Similar to the detection sensitivity of EPID-based 2D in vivo dosimetry [8], EPID-based 3D in vivo dosimetry performed using planning CTs was sensitive to the machine output, MLC shift and PTV deformation variations, but has a lower sensitivity to Phantom shift and gantry and couch angle shift variations. Different from the way of evaluating the gamma results between the EPID reconstruction and the TPS calculated dose for each variation [8, 26], this study chose to evaluate the gamma consistency between the EPID reconstruction and TPS simulation. The advantage is that not only can the detection sensitivity of the EPID reconstruction be quantitatively analyzed, but the limitations of EPID-based 3D in vivo dosimetry performed using planning CTs can also be found.

Similar to previous works [27, 28], strong correlations between the detection sensitivity and PTV DVH indicators were also found in our study (Figs 6–8). Of course, the main purpose of using DVH indicators in this study was to validate the effect of variations on the accuracy of the EPID reconstruction dose, which has not been reported in other studies. Our simple phantom results show that the DVH trend between the EPID reconstruction and TPS simulation is consistent only for the machine output and MLC shift variations. This finding indicates that only the machine output and MLC shift will not affect the reconstruction dose accuracy of the EPID-based 3D in vivo dosimetry performed using planning CTs. The machine output and MLC shift are phantom-unrelated variations that cause changes in the beam of the linear accelerator. Therefore, it can be determined that EPID-based 3D in vivo dosimetry can effectively detect phantom-unrelated variations. Using a 2%/2 mm gamma standard, a 3% machine output variation and 1-mm MLC shift variation can be detected, meeting the requirements of the AAPM Task Group report 142(TG142) [29] for the machine output (daily check) and MLC tolerance.

Phantom shift variations and gantry/couch angle shifts are phantom-related variations and may cause the exposure site to deviate from the planned target area. EPID reconstruction performed using planning CTs will lose this important position shift information, and the sensitivity can only depend on the uniformity of the tissue density at the exposure site (Fig 5). These limitations not only reduce the detection sensitivity of EPID-based 3D in vivo dosimetry for phantom-related variations but also affect the accuracy of the reconstructed dose (Fig 7). We further validated the effect of the Phantom shift on the dose accuracy of the EPID reconstruction by a thoracic phantom and proved that CBCT can guide the accurate EPID dose reconstruction (Fig 9B). The maximum clinically acceptable setup error is 5 mm [30]. The results of the thoracic phantom (Table 3 and Fig 9) highlight that EPID-based 3D in vivo dosimetry performed using planning CTs may not be a reliable clinical analysis system for phantom-related variations, but CBCT-guided EPID reconstruction provides a more reliable clinical assessment for both PTV and OARs.

EPID-based 3D in vivo dosimetry performed using planning CTs has a high sensitivity to PTV deformation (Fig 3), but the accuracy of the EPID reconstruction dose will be affected (Fig 8), especially for locally heterogeneous sites.

Anatomical changes usually occur during treatment, and the planning CT will limit the accuracy of the EPID reconstruction dose. CBCT as online images acquired during treatment may be more suitable for the in vivo verification of anatomical changes if accurate CBCT dose calculations are available [31, 32].

## Conclusion

EPID based EPID-based 3D in vivo dosimetry performed using planning CTs is sensitive to the variations, that are phantom-unrelated and cause changes in the beam of the linear accelerator, such as machine output and MLC shifts. Planning CTs will limit the detection sensitivity and the accuracy of the reconstruction dose of the EPID-based 3D in vivo dosimetry for phantom-related variations, such as Phantom shift and gantry/couch angle shift. EPID reconstruction combined with IGRT technology has been proven to be a more effective method to monitor phantom shift variation.

## Supporting information

S1 Fig The dose distribution for the thoracic phantom plan. (a) transverse. (b) sagittal.
(TIF)

S2 Fig Dose comparison of EPID reconstruction and TPS for the original IMRT plan delivered to thoracic phantom. (a) Isodose distribution, EPID reconstruction (dashes) and TPS (solid). (b) 2%/2 mm gamma distribution, γ>1 (red), γ≤ 1(blue).
(TIF)
