# Supplementary figures and images for: Investigating the effectiveness of monitoring relevant variations during IMRT and VMAT treatments by EPID-based 3D in vivo verification performed using planning CTs
Source: PLoS One. 2019 Jun 28;14(6):e0218803. doi: 10.1371/journal.pone.0218803 (PMC6599132; doi:10.1371/journal.pone.0218803)

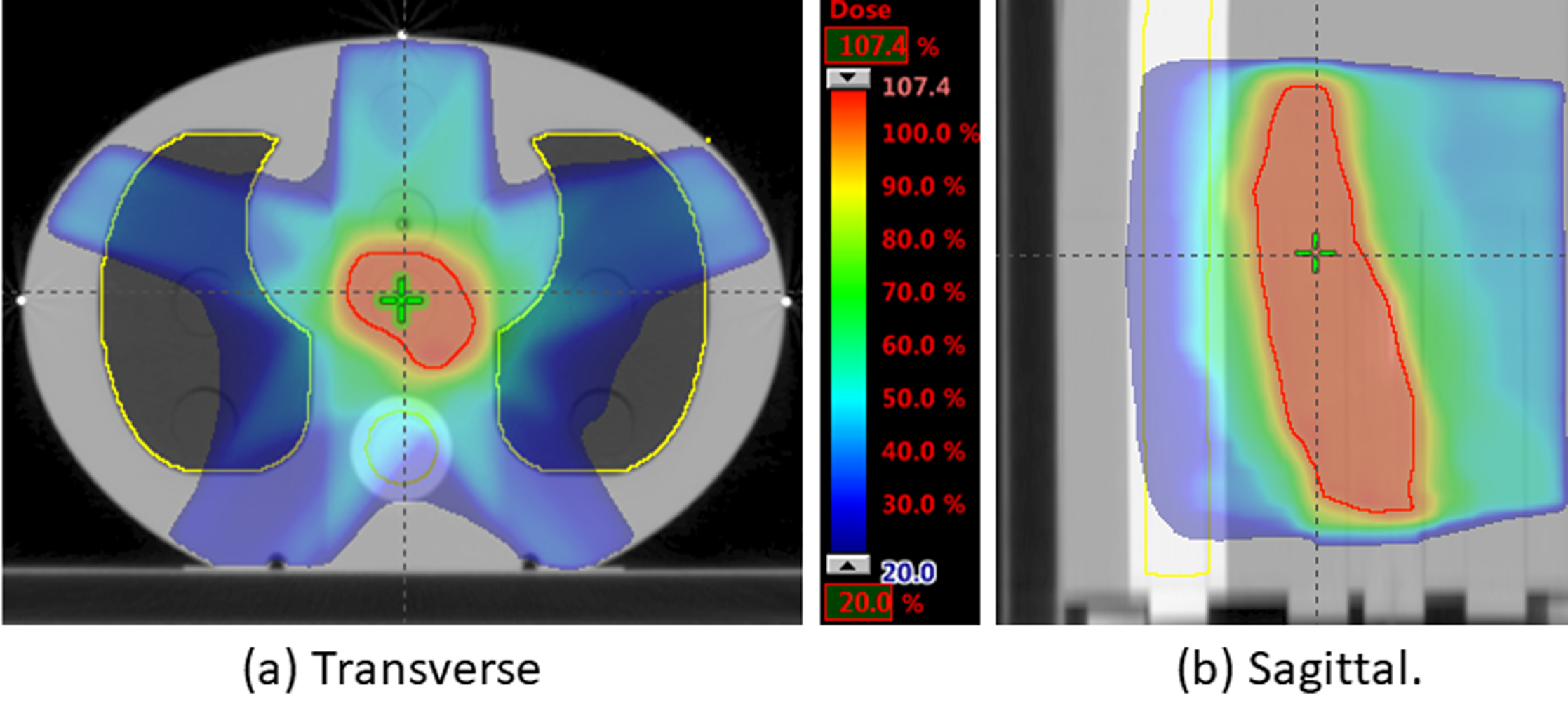

Supplement: S1 Fig — (a) transverse. (b) sagittal. (TIF) [file pone.0218803.s001.tif]

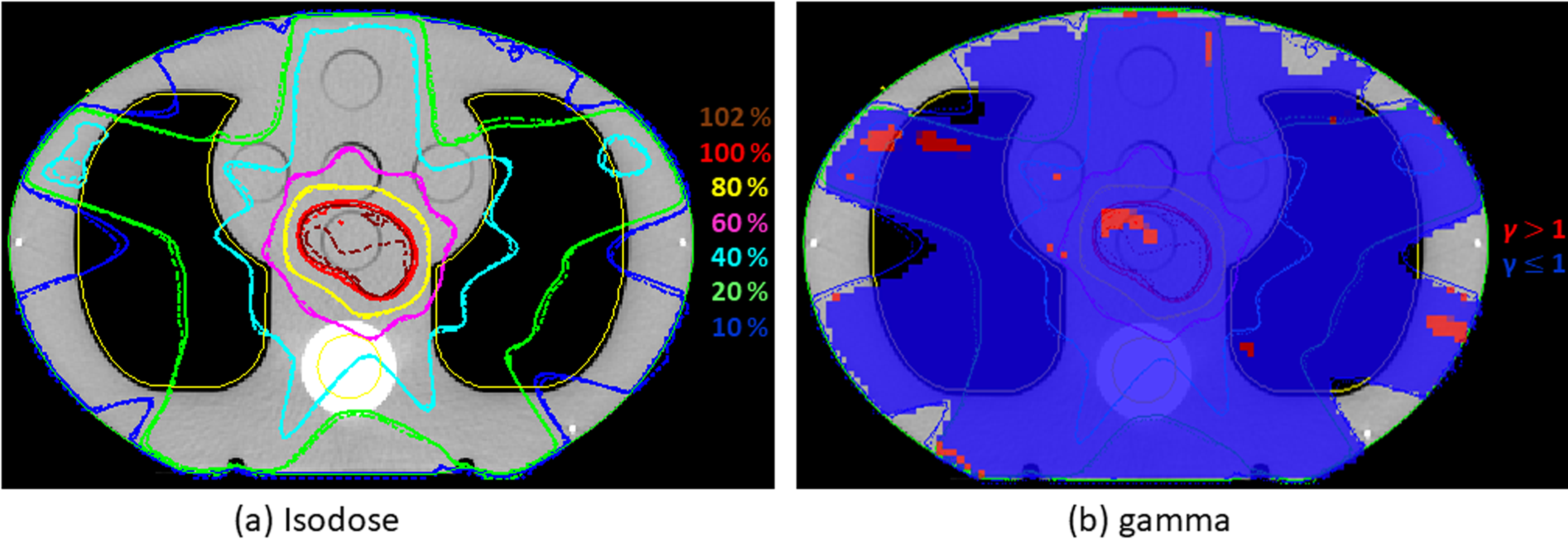

Supplement: S2 Fig — (a) Isodose distribution, EPID reconstruction (dashes) and TPS (solid). (b) 2%/2 mm gamma distribution, γ>1 (red), γ≤ 1(blue). (TIF) [file pone.0218803.s002.tif]
